# Micro-to-Nanometer Scale Patterning of Perovskite Inks via Controlled Self-Assemblies

**DOI:** 10.3390/ma15041521

**Published:** 2022-02-18

**Authors:** Misun Kang, Dooho Choi, Jae Young Bae, Myunghwan Byun

**Affiliations:** 1 Department of Advanced Materials Engineering, Keimyung University, Daegu 42601, Korea; misun.kang@gmail.com; 2 Department of Chemistry, Keimyung University, Daegu 42601, Korea; 3 School of Advanced Materials Engineering, Dong-Eui University, Busan 47340, Korea; dhchoi@deu.ac.kr

**Keywords:** perovskite inks, controlled self-assemblies, coffee ring effect, Marangoni flow, micro-to-nanoscale patterning

## Abstract

In the past decade, perovskite materials have gained intensive interest due to their remarkable material properties in optoelectronics and photodetectors. This review highlights recent advances in micro-to-nanometer scale patterning of perovskite inks, placing an undue emphasis on recently developed approaches to harness spatially ordered and crystallographically oriented structures with unprecedented regularity via controlled self-assemblies, including blade coating, inkjet printing, and nanoimprinting. Patterning of the perovskite elements at the micro- or nanometer scale might be a key parameter for their integration in a real system. Nowadays, unconventional approaches based on irreversible solution evaporation hold an important position in the structuring and integration of perovskite materials. Herein, easier type patterning techniques based on evaporations of polymer solutions and the coffee ring effect are systematically reviewed. The recent progress in the potential applications of the patterned perovskite inks is also introduced.

## 1. Introduction

For the last few decades, lithography techniques in the semiconductor industry are dramatically developed because it is essential for the critical dimension (CD) of the integrated circuit to be extremely minimized from a few hundred nanometers to a few nanometers [1]. This significant progress of nanolithography in the semiconductor industry includes inheritances of successive paradigm shifts to overcome the diffraction limit, which can define the resolution of an optical system. To fabricate integrated circuits (ICs) with a certain CD smaller than the resolution of the lithography system, multiple patterning techniques are widely used [1,2]. This method is based on multiple steps of the lithography process and etching process with a finite lateral shift (equal to CD), thus opening a new path to fabricate 10 nm and 7 nm node semiconductor processes and beyond. As an alternative method to fabricate sub 10 nm scale IC architectures, an extremely ultraviolet (EUV) lithography system using a wavelength of 13.5 nm (almost X-ray range) was developed in the past few years [3,4]. Since the resolution limit of the photolithography system is directly proportional to the wavelength and inverse proportional to the numerical aperture of the system, the controllable EUV can give us a great possibility to reach sub-7 nm node semiconductor processes with multiple patterning techniques. 

In general, lithography techniques are categorized into two individual types: lithography with or without a mask (referred to as “reticle”). The former techniques, including photolithography [5,6], soft lithography [7], and nanoimprint lithography [8,9], enable easier access to transferring micro-to-nanometer scale patterns over a large area, thus leading to a high-throughput fabrication process, whereas the latter techniques, including electron beam lithography [10,11] and focused ion beam lithography [12], are applied to fabricate arbitrary patterns using programs, which can control the systems. Particularly, these techniques have good fit for the fabrication of ultrahigh-resolution patterns and arbitrary shapes with minimum feature size as small as a few nanometers. However, the production speed is limited, and it is inappropriate for high-throughput mass production. Contrary to the numerous advantages of nanolithography techniques as mentioned above, these methods contain intrinsic disadvantages. First, lithography is expensive to fabricate ICs in a large area because a huge and extremely expansive lithography machine is necessary, which depends on the size of the wafer (scientifically referred as to “substrate”). Moreover, most lithography techniques should use “resists” which react with the incident lights or electron beams. Since the surface of substrates after coating with resists should be clearly protected by dust or small particles, a tremendously expensive, wide, and environmentally controlled cleanroom is mandatory for the use of most lithography methods. Second, lithography is harmful since most chemicals such as photoresists, e-beam resists, and chemicals for cleaning and etching in semiconductor manufacturing areas are known to be toxic. Therefore, a brand-new lithography method, which can fabricate distinguishable and controllable patterns in a large area with extremely low cost, is strongly required for the next-generation semiconductor fabrication process. Furthermore, this approach can be potentially applied for manufacturing high-performance optoelectronics devices and photodetectors in a cost-effective manner.

Recently, perovskite-based solar cells have received considerable attention due to their excellent optoelectronic properties, including an excellent light absorption coefficient, tunable bandgap, long charge diffusion length, and high carrier mobility [13,14,15,16]. Based on these outstanding optical properties, the efficiency of lead halide perovskite film photovoltaics was reported to be 25.5% in 2020 [17,18]. Moreover, organic or inorganic perovskites are widely exploited in the research areas of lasers [19,20], nonvolatile memories [21], field-effect transistors [22], photocatalysts [23,24,25], and so on. Importantly, devices with high efficiencies were obtained through solution processing which is a facile and large area coverage method.

In this Review, we introduce easier type patterning techniques based on evaporations of polymer solutions and the coffee ring effect and its various applications. As a ubiquitous phenomenon, the evaporation of polymer droplets, including nonvolatile solutes, shows contact line pinning and the formation of ring-like residues, which is so-called “coffee-strains” or “coffee-ring” [26,27]. Understanding and utilizing the coffee ring effect is quite important to fabricate the ordered structure because it is an alternative way to quickly make successive and identical patterns with extremely low cost [28]. The conventional patterning processes are following: (i) thin film deposition, (ii) resists coating and lithography process, (iii) etching resists and thin film, and (iv) removing residues and cleaning surface. These complex fabrication procedures need a long time and expensive facilities. However, in the patterning process based on the coffee ring effect, polymer solution droplet process and evaporation nature contain all complicated processes as mentioned above. Herein, easier type patterning techniques based on evaporations of polymer solutions and the coffee ring effect are systematically reviewed. The recent progress in the potential application of patterned perovskite inks is also introduced.

## 2. Coffee Ring Effect and Marangoni Flow

**Figure 1:**
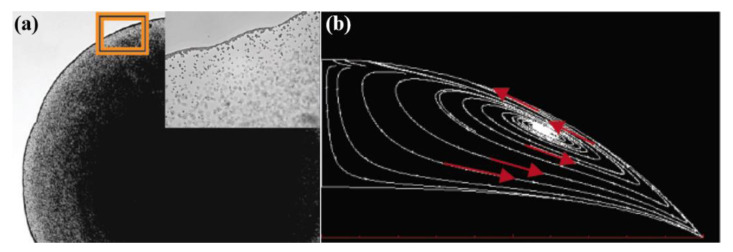
(**a**) Five times magnification image of a droplet with 0.5 wt% PS particles at 0.05 times after instillation. The inset is at 63× magnification of the particles gathered in the sphere line. Reproduced with permission [30]. Copyright 2012, American Chemical Society. (**b**) Theoretical flow field of the octane droplet showing Marangoni flow. Reproduced with permission [33]. Copyright 2006, American Chemical Society.

In 1997, Deegan et al. reported that a drop of coffee evaporating over a plate leaves a ring-shaped residue, a so-called “coffee ring”, which is derived from the capillary flow of irreversible solvent evaporation [26,27,29]. Dr. Yodh’s group took snapshot images of a droplet with 0.5 wt% PS particles in the middle of evaporation shown in Figure 1a [30]. At 0.05 s, after the droplet formed, the particles were washed up on the edge line by the capillary force [30]. The evaporation at the edge of the drop is faster, and it carries particles outwards, resulting in a ring-shaped stain [29,31,32]. The outward capillary flow interferes with a homogenous, uniform deposit for inkjet applications. To solve the problem of inhomogeneous stains caused by the coffee ring effect, various methods to relieve the coffee ring effect caused by the temperature gradient driven Marangoni flow are being studied using surface tension gradient [31,33], electrowetting to make the surface hydrophobic [34], acoustic streaming with surface acoustic waves [35], etc. Dr. Hu studied the Marangoni effect with a droplet of PMMA fluorescence particles in octane and other alkanes by controlling the temperature of substrates. Marangoni flow can be observed in several solvent mixtures and is discovered in red wine, which involves the evaporation rate difference in water and alcohol. When the wine is shaken in a glass, evaporation first begins on the wall of the glass. Alcohol evaporates faster than water, and this is much faster in the thin layer that forms on the glass wall. As the alcohol evaporates from the thin layer of wine, the alcohol concentration decreases (the water concentration increases), so the surface tension inside it becomes higher than the surface tension in the center of the glass. Therefore, the wine climbs until reaching the top of the thin layer and falls due to gravity. This was named tears of wine by James Thomson in 1855 [28] and is defined as the Marangoni effect. In Figure 1b, the theoretical flow field is described, and it agrees well with the experiment [33]. This flow leads to the circulation of a solution inhibiting the accumulation of solutes.

## 3. Various Approaches for Patterning of Perovskite Inks

### 3.1. One Step Solution Coating

**Figure 2:**
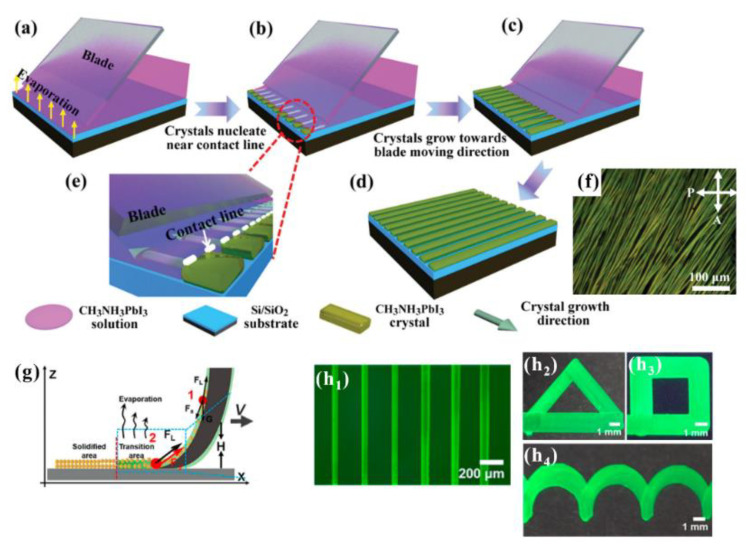
Schemes of one-step blade coating methods (**a**) the perovskite solution is dragged with a blade to form a triple-phase contact line. (**b**) The crystal nucleates close to the contact line. (**c**) Along the movement of the blade, the nuclei develop into crystals. (**d**) The CH_3_NH_3_PbI_3_ arrays are created parallel to the moving direction of the upper blade. (**e**) Enlarge image of (**b**). (**f**) Cross-polarized optical image of the scheme (**d**). Reproduced with permission [45]. Copyright 2020. Wiley-VCH. (**g**) Fine-tuned conical fibers, called “Chinese brush”, transfer QD solutions onto the substrate. (**h**) Various QD patterns drawn by the Chinese brush are shown: 40 µm-width line arrays (**h_1_**), a triangle (**h_2_**), a square (**h_3_**), and a wave (**h_4_**). Reproduced with permission [44]. Copyright 2018, American Chemical Society.

The flow coating method reported in 2014 by Dr. Register was introduced to form thin polymer film using polymer solutions [36,37,38,39]. According to the paper, the thickness of the polymer films having less than 200 nm is affected by the solution volume of the reservoir, the angle between a blade and a substrate, gap height, etc. Several groups tried to apply this approach to fabricate smooth, uniform, and thin perovskite crystal films [40,41,42]. Recently, perovskites have emerged as a promising material with the development of optoelectronic device technology or photodetector fields. In addition, to improve device stability and high responsivity, the spatial formation of one dimensional (1D) structures at the micro-to-nanometer scale is of critical importance. Therefore, trials for arrays or patterns via the blade coating of the perovskite solution are reported [43,44]. Dr. Jie verified the one-step alignment of CH_3_NH_3_PbI_3_ microwires in Figure 2f, and the formation process of CH_3_NH_3_PbI_3_ microwires is shown in Figure 2. As a blade was dragged from the front of the substrate with the perovskite solution, solvent evaporation was initiated, and the triple-phase contact line was formed from the trapped solution (see Figure 2a). During the solvent evaporation, solutes (perovskite crystals) are precipitated along the contact line and grow up to microwires in the same direction as the blade moving in Figure 2b,c. Figure 2e show an enlarged view of the contact line and the blade moving in one direction. Figure 2d represent the aligned 1-D CH_3_NH_3_PbI_3_ microwires on the whole substrate [45]. 

Another trial of one-step micropatterns for the applied display was suggested by Dr. Jiang and coworkers called “Chinese brush”, in 2015. The Chinese brush method uses finely controlled brush-coating or tuned conical fibers to transfer quantum dot solution directly onto a substrate without a template to create various micro patterns in Figure 2(h_1_–h_4_). As the Chinese brush moves at a constant speed, the solution is kept stable within the fibers, and the triple-phase contact line formed from small meniscus curves is also moved (Figure 2g). The movement of the triple-phase contact line controlled by the velocity of conical fibers is balanced between the cooperative effect of the Marangoni flow of quantum dot (QD) solution and the Laplace pressure given by conical fibers. Brushing can then be programmed in the three-axis motion stage via specific software, and various patterns are fabricated as shown in Figure 2(h_1_–h_4_) [44].

### 3.2. Meniscus Assisted Solution Printing

**Figure 3:**
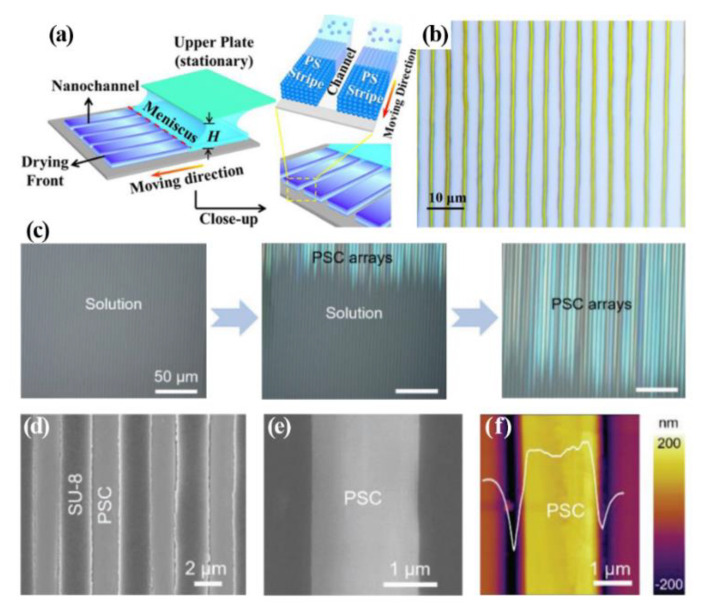
(**a**) Scheme of meniscus−assisted solution-printing to build nanochannels between PS stripes (**b**) Representative image of perovskite single-crystal arrays. Reproduced with permission [46]. Copyright 2020. Wiley-VCH. (**c**) The changing process from the bulk solution to perovskite crystal arrays is due to capillary force after filling the solution into microchannels. (**d**) SEM image of the CH_3_NH_3_PbI_3_ arrays. (**e**) Magnified SEM image of (**d**). (**f**) AFM image of one line of perovskite crystal arrays. Reproduced with permission [43]. Copyright 2016. Wiley-VCH.

The meniscus-assisted solution printing (MASP) method used to synthesize the ordered various polymer and perovskite arrays using capillarity was reported by Dr. Lin and his group [46,47,48,49]. The MASP technique, which uses a moving substrate and a stationary blade, begins using prepared microchannels via a polystyrene (PS) latex nanoparticles aqueous suspension as a space confiner for capillarity shown in Figure 3 [46]. Perovskite solution is in between the substrate with microchannels and a blade, and some of the solution is filled into the microchannels as the substrate moves. Subsequently, the deposited solution is converted into perovskite crystal arrays through nucleation and growth on solvent evaporation. At the edge of the meniscus, the three-phase contact line was moving faster than the center during evaporation; therefore, the perovskite crystal arrays formed tidily while dragging the solution by the blade. Dr. Zhang’s group also published a paper about perovskite crystal arrays via blade coating within photoresist channels made by photolithography [45]. Figure 3c show the changing process of the perovskite solution on the substrate with the photoresist channels as the blade is dragged. The last figure in Figure 3c is the middle of solvent evaporation which means the perovskite crystal arrays were forming. From the SEM images of Figure 3d,e, the microchannels were filled with perovskite solution, changing into perovskite crystal arrays. Figure 3f also reveal that the crystal arrays have no grain boundary or defects and have a roughness of 4–6 nm on the surface.

## 4. Inkjet Printing Method

One of the most frequently used solution processes is inkjet printing, which can fabricate fine patterns on various substrates, including a flexible film. There are several methods for inkjet printing, such as continuous inkjet printing, drop-on-demanded inkjet printing, etc. [50]. First, a continuous inkjet has a constant flow under the summation between a charge potential and gravity. The biggest advantage is that there are no restrictions on the substrates but there is an unavoidable waste of ink. Next, the drop-on-demand inkjet printing method controls the drop accurately, but there is a restriction of solution viscosity. For all inkjet printing methods, the coffee ring effect affects the evaporation after the ink is dropped on a substrate [50].

### 4.1. Control the Coffee Ring Effect by Several Factors: Adding Polymer or Adjusting Temperature of a Substrates

Although inkjet printing is a non-contact, material-effective, and large-area applicable method in the solution-processing technique, the drying process is still challenged. The evaporation and crystallization processes of the solution as an ink after being dropped cannot be controlled. The coffee ring effect, inherent property in solution drying, affects the surface uniformity of the perovskite patterns or dots [51,52,53]. Therefore, controlling the crystallization rates of the solution can be a key factor in controlling crystal size. 

**Figure 4:**
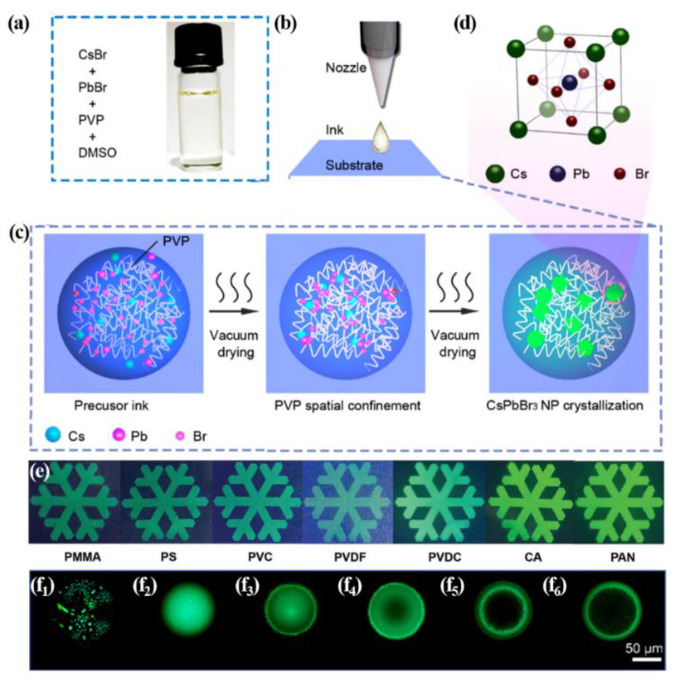
(**a**) CsPbBr_3_/PVP/DMSO solution for inkjet printing (**b**) the process of inkjet printing: the moment the CsPbBr_3_/PVP/DMSO solution comes out from nozzle (**c**) schematic diagram of evaporation and crystallization after dropping ink. (**d**) final lattice structure of CsPbBr_3_ Perovskite crystal. Reproduced with permission [51]. Copyright 2019, American Chemical Society. (**e**) Optical images of inkjet printing with MAPbBr_3_ perovskite and various polymer solutions. Reproduced with permission. Copyright 2019. Wiley-VCH [53]. (**f**) Photoluminescence microscope photographs of single dot crystallized in an ambient environment (**f_1_**), vacuum-dried at 20° (**f_2_**), 30° (**f_3_**), 40° (**f_4_**), 50° (**f_5_**), and 60° (**f_6_**) as the substrate temperatures. The scale bar is 50 µm. Reproduced with permission [51]. Copyright 2019, American Chemical Society.

To control the coffee ring effect and evaporation rate, Dr. Kim and coworkers suggested using a long-chain polymer named polyvinylpyrrolidone (PVP) in perovskite precursor solution for inkjet printing [51]. In Figure 4a, the perovskite precursor solution consists of adding CsBr, PbBr_2_, and PVP into dimethyl sulfoxide (DMSO) solvent. When the ink is dropped onto the substrate, the precursor materials and PVP are mixed randomly in a droplet. The long chains in the PVP give viscosity and spatial confinement for solutes which are precursor materials and perovskite nanoparticles, so the solutes are retain uniform distribution during DMSO evaporation and perovskites’ crystallization shown in Figure 4c. The cubic lattice in Figure 4d is the final structure after crystallization. Other ways to adjust the evaporation rate of the solvent and crystallization processes are controlling the polymer concentration, mixing the solvents, or heating/cooling during the processes [51]. These methods are ways of controlling the flow inside the droplet or solvent movements. Li et al. show the different fluorescence microphotograph images depending on PVP concentrations from 100, 200, 300, 400, and 500 mg·mL^−1^. Higher PVP concentration increases the uniformity and brightness and reduces the coffee ring effect [52]. This is because the higher the PVP concentration, the higher the viscosity and the more spatially restricted the capillary flow. Changing the type of polymer to the perovskite precursor solution has similar results in inkjet printing as well. In Figure 4e, polymethyl methacrylate (PMMA), polystyrene (PS), polyvinyl chloride (PVC), polyvinylidene fluoride (PVDF), polyvinylidene chloride (PVDC), cellulose acetate (CA), and polyacrylonitrile (PAN) are each utilized as the polymer film surfaces onto which the perovskite precursor solution is dripped. The droplet of the perovskite precursor solution interacts differently with the polymers of the surface depending on their molecular length, weight, or shape. The perovskite dot-printed images made by the droplets were able to peel off and then tested for resistance to water. The perovskite dot image on PVDC showed stable luminescence for 100 days in water, whereas the perovskite dot patterns on other polymer films (PMMA, PS, PVS, PVDF, PAN and CA) were began degradation after 20, 50, 4, 1, 1.5, and 0.1 h later, respectively [54]. Meanwhile, although the polymer is mixed in the precursor solution, the perovskite crystals are sparsely formed when the droplets evaporate in the ambient environment in Figure 4(f_1_). This is because the evaporation rate is slow, and the perovskite nuclei developed before the space constraints occurred. To increase the evaporation rate, vacuum drying and heating on the substrate are performed simultaneously. Perovskite crystal with a bright, uniform surface is shown in Figure 4(f_2_) after vacuum drying at 20 °C. Other results from 30, 40, 50, and 60 °C heating on the substrate in a vacuum can be found in Figure 4(f_3_–f_6_), respectively [51]. As a result, if the evaporation is too fast, the coffee ring effect occurs again. This indicates that vigorous capillary flow occurs when heat is applied to the substrate.

### 4.2. Control over the Coffee Ring Effect by Several Factors: Mixing Solvents or Confined the Area

**Figure 5:**
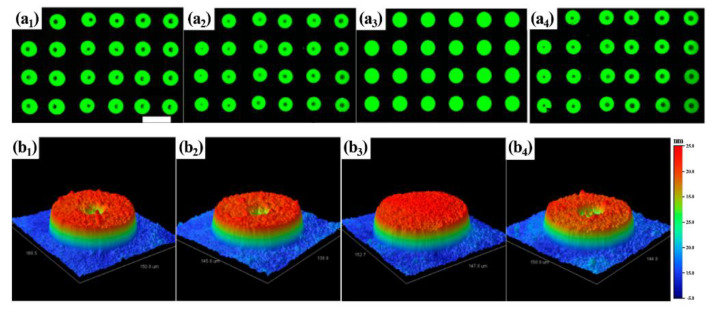
(**a**) Fluorescence microscope images of QD dot films (scale bar is 200 µm). The precursor inks have oDBC of 0 (**a_1_**), 10 (**a_2_**), 20 (**a_3_**), and 30% (**a_4_**) by volume, respectively. (**b_1_**–**b_4_**) 3D morphology images of (**a_1_**–**a_4_**), respectively. Reproduced with permission [54]. Copyright 2019, American Chemical Society.

Another way to reduce the coffee ring effect is to control Marangoni flow by mixing solvents. In Figure 5(a_3_,b_3_), Dr. Cao and his group obtained uniform and flat surface dotlike film with quantum dots (QDs) in a 20 vol% 1,2-dichlorobenzene (oDCB) and cyclohexylbenzene (CHB) mixture by inkjet printing [54]. Pure CHB has a viscosity of 3.68 cP and surface tension of 34.5 mN/m, but after the dispersion of QDs in CHB, the viscosity decreases to 3.14 cp and the surface tension increases to 41.31 mN/m because of the interaction between CHB and ligands of QDs. The resulting film, using only CHB as a precursor ink, is shown in Figure 5(a_1_,b_1_), in which the coffee ring effect occurred. By adding 10% and 20% vol of oDCB with a viscosity of 1.33 cP and surface tension of 36.6 mN/m to the QD ink of CHS, the viscosity and the surface tension of the ink are reduced, and the coffee ring effect is moderated (see Figure 5(a_2_,b_2_)). The 20% vol oDCB in a precursor ink formed uniform and smooth surface dots without the coffee ring effect in Figure 5(a_3_,b_3_). However, when the 30% vol of oDCB was reached, the viscosity and the surface tension increased again, and the surface of the resulting dots showed the coffee ring effect (see Figure 5(a_4_,b_4_)). This is because the viscosity and surface tension appropriately restrained the motion of the solutes. Dr. Sun and his group prepared an ink of a perovskite precursor solution using a mixed solvent of toluene (TOL) and dodecane (DOE) for inkjet printing to fabricate microarrays [55]. The photoluminescence microscopic images from a perovskite drop with a mixed solvent are due to the different ratios of DOE to TOL, which causes the degree of the coffee ring structure. The Photoluminescence (PL) image with a volume ratio of DOE to TOL of 6:4 shows a uniform, bright surface that is hardly affected by the coffee ring effect. This is because Marangoni flow is caused by the difference in the boiling temperature of the solvents, and DOE and TOL have 215 °C and 110 °C as boiling points, respectively. The researchers explained how the difference in boiling temperatures affects the Marangoni flow. The droplet edge areas of the perovskite precursor solution dissolved in one solvent evaporate first, so Marangoni flow causes solute migration from the low to the high surface tension area. The usage of a mixed solvent with different boiling points makes the coffee ring effect weakened. In the droplet edge area of the precursor ink, the low-boiling solvent evaporates first, leaving behind the high-boiling solvent. Meanwhile, in the top area of the droplet, there is still a mixed solvent of TOL and DOE. Because the surface tension of the DOE-perovskite solution is smaller than that of the TOL-perovskite solution, the surface tension of the edge region is lower than that of the top region (the top region has a mixed solvent of TOL and DOE). Marangoni flow causes the low surface tension in the edge area to flow to high surface tension in the top area, and balances it with the capillary flow, resulting in a uniform surface [55]. Kim et al. [56] specifically investigated a way to relieve the coffee ring effect using Marangoni flow from binary mixed solvents with different boiling points. The results also included the effects of different environments in opened and closed areas on the coffee ring effect during evaporation. As mentioned above, the evaporation of mixed solvents with different boiling points causes Marangoni flow to moderate the coffee ring effect. The researchers used water-soluble quantum dots from ZEUS in Korea to confirm the results. The coffee ring effect was not completely removed using only the mixed solvent, but it was alleviated. Marangoni flow is maintained for a short time only while ethanol evaporates first but lasts longer during evaporation in a confined chamber. This is because the entrapped ethanol vapor near the droplet after evaporation maintains a surface tension gradient for Marangoni flow [56]. 

**Figure 6:**
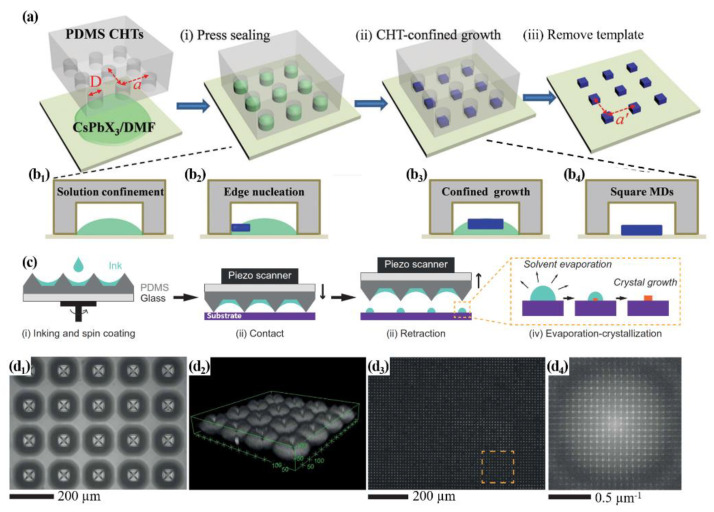
(**a**) Schematic illustrations of the process for fabrication of the micro-disk arrays in CHT-confined area. (**b**) The enlarged images of (**a**_(**i**)_,**a**_(**ii**)_); from the nucleation to final nanocrystals (**a**_(**iii**)_) of CsPbX_3_ perovskite. Reproduced with permission [58]. Copyright 2017. Wiley-VCH. (**c**) Another fabrication method for perovskite nanocrystal arrays with PDMS templates called Polymer pen lithography (PPL); the injection of precursor solutions of MABr and PbBr_2_ dissolved in DMSO into the Piezo scanner (**c**_(**i**)_), contact the scanner on a substrate (**c**_(**ii**)_), detach it and leave droplets of the precursor solutions (**c**_(**iii**)_), and evaporation and crystallization of the perovskite nanocrystals (**c**_(**iv**)_). (**d_1_**) A optical micrograph of the precursor solution around the base pyramid in the scanner. (**d_2_**) 3D confocal microscopic image of precursor ink with dye after scanner contact on the substrate. (**d_3_**) Fluorescence micrograph of MAPbBr_3_ nanocrystal arrays. (**d_4_**) Fourier transform of orange-dot-square in (**d_3_**). Reprinted with permission [59]. Copyright 2020, American Association for the Advancement of Science.

Halide perovskite nanocrystal arrays evaporated in a confined area are shown in Figure 6a. Dr. Fu and his group used CsX and PbX_2_ in dimethylformamide (DMF) as a precursor solution to fabricate CsPbX_3_ (X = Br, Cl, or mixed one) halide perovskite micro-disk arrays. They first prepared a polydimethylsiloxane (PDMS) cylindrical hole template (CHT) by a photolithography method and press-sealed it to a substrate (see Figure 6(a_(i)_,b_1_)). Then, the precursor solution was injected into the empty space between the CHT and the substrate. During the evaporation of DMF, perovskite nucleated at the edge region of the CHT and grew rapidly (see Figure 6(a_(ii)_,b_2_,b_3_)). As mentioned earlier, the evaporation of the solvent during the nucleation and growing of the perovskite nanocrystal in the confined area relieves the coffee ring effect, and it helps to make uniform and regular nanocrystal arrays [57]. The elimination of the PDMS-CHTs is the final step for micro-disk arrays fabrication with the same distance of the templates (a’) shown in Figure 6(a_(iii)_,b_4_) [58]. Another way of using a PDMS template to synthesize nanocrystal arras was published by Dr. Dravid and Dr. Mirkin [59]. They synthesized perovskite nanocrystal arrays on large-area substrates using the polymer pen lithography method in conjunction with PDMS pyramidal pens. As shown in Figure 6c, the precursor solutions were spun onto the PDMS template, and the solution, as ink, was stagnated around PDMS pyramidal pens because of high surface tension and low viscosity of the solution shown in Figure 6(d_1_,d_2_). As shown in Figure 6(d_3_,d_4_), the well-ordered periodic nanocrystal array is displayed over the entire substrate. The authors controlled the size of the nanocrystal by the initial precursor concentration and the extension length of the PDMS pyramidal pens. As predicted, a thicker concentration fabricated larger crystals [59]. Song et al. [60] reported that the control of the adhesion of a substrate plays an important role in the fabrication of perovskite single-crystal microplate arrays. They prepared various substrates with different adhesive forces such as bare-Si wafer (0.75 mN), silicon modified with (3-glycidoxypropyl)trimethoxysilane (GPTS-Si, 0.65 mN), silicon modified with Triethoxy(octyl)silane (TOS-Si, 0.58 mN), silicon modified with 1H,1H,2H,2H-perfluorodecyltrimethoxysilane (PFOS-Si, 0.53 mN), PDMS modified with PFOS (PFOS-PDMS, 0.55 mN), FTO attached PFOS (PFOS-FTO, 0.62 mN), and PFOS-Micro-nanostructured Al (0.74 mN) (in supporting information of [60]). The pre-materials of perovskite in the substrate with a low adhesion tended to stay in the center during evaporation and formed crystals in the center of the droplet. However, on the high adhesion substrate, the solutes seemed to be affected by the coffee ring effect due to the long time pinned at the contact line. Finally, the perovskite crystal microarrays successfully formed on PFOS-Si and PFOS-PDMS substrates with low adhesion. 

### 4.3. Control the Coffee Ring Effect by Several Factors: Particle-Shape Effects

**Figure 7:**
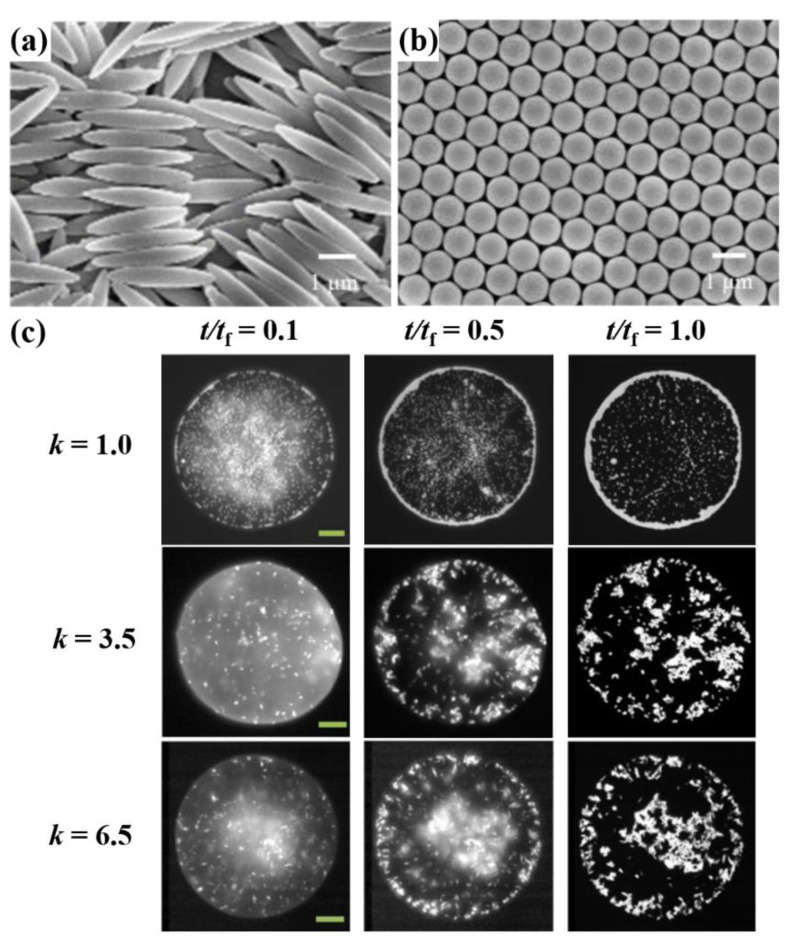
(**a**) A scanning electron microscopy (SEM) image of elliptical PS particles with an aspect ratio of *k* = 6.5. (**b**) An SEM image of spherical PS particles. (**c**) The microscopic images of the different shaped (*k* = 1, spheres; *k* = 3.5; *k* = 6.5 for aspect ratio) particle migration after colloidal drop of 0.5 wt%. All scale bars are 20 µm. Reproduced with permission [63]. Copyright 2016, American Chemical Society.

Yunker et al. [61] reported particle-shape effects in solution in 2011. They employed micrometer-sized polystyrene particles with different shapes in water. The spherical (*k* = 1.0) particles in the water droplet gathered near the rim area as they evaporated, called the coffee ring effect. On the other hand, the ellipsoidal particles (*k* = 3.5), using the same polymer compound and in the same solvent, only migrated until they arrived at the air–water interface. Then, after they dried up, the loosely packed state was caused by the interparticle attraction between anisotropic particles, which is more than two orders stronger than the attraction between spherical particles [61,62]. Kim et al. [63] also showed the particle shaped effect with spherical (*k* = 1) and two different ratios of aspect ratio (*k* = 3.5 and *k* = 6.5). The SEM images of the spheres (*k* = 1) and ellipsoidal PS particles (*k* = 6.5) are shown in Figure 7a,b. Figure 7c represent the evaporation process of a droplet of the inkjet ink with three types of particles. The spherical particles tended to migrate from edge to edge at *t*/*t_f_* = 0.1 and 0.5, and eventually, the particles gathered to the edge lines after evaporation was complete. Meanwhile, the ellipsoidal particles stayed in the middle until *t*/*t_f_* = 1.0. The spheres continuously migrated to the edge of the droplet, whereas the ellipsoidal particles occasionally moved, and others stayed in the middle of the droplet [63]. In previous studies, a method of forming the various shapes of perovskite materials was reported [64]. Following this method, the shape-controllable perovskite crystals can be used to realize the particle-shape effects.

## 5. Imprinting Method and Others

### 5.1. Imprinting Method

**Figure 8:**
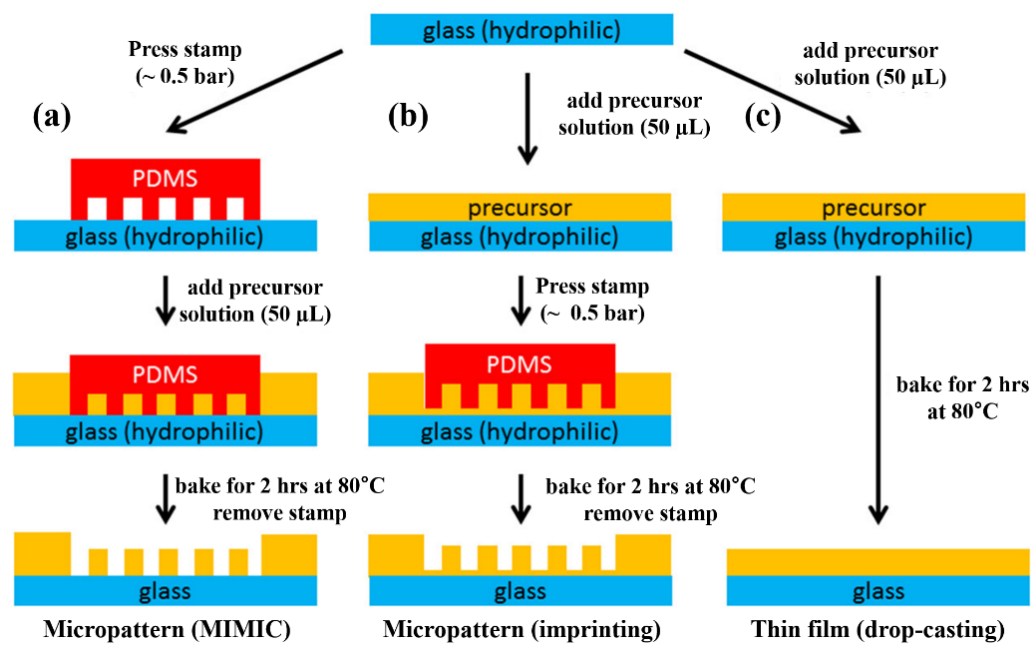
Schematic illustrations of two ways for micropatterning methods and drop-casting method for films as a reference: (**a**) MIMC method, (**b**) imprinting lithography method, and (**c**) drop-casting method. Reproduced with permission [66]. Copyright 2018, American Chemical Society.

Nanoimprinting is renowned for the fabrication of depositions on large or complicated substrates with low cost, high resolution, and high throughput. Garnett et al. [65] successfully manufactured perovskite pattern deposition with uniform coverage in which the perovskite pattern crystallized during processing. The evaporation of the solvent in the pre-annealing steps enabled the precursor solution supersaturation state to initiate nucleation for perovskite crystallization. They also investigated how solvents affected the morphology of the perovskite crystal gratings with DMSO and DMF. Because DMF evaporates faster than DMSO, using DMSO in the precursor solution can obtain micro-sized and smooth surface crystals, and the precursor solution in DMF undergoes rapid evaporation resulting in isolated crystals (not smooth forms). A mixture solution of DMSO and DMF (4% by volume of DMSO) produced regular perovskite crystal gratings after the imprinting process, and the film after spin-coating during the imprinting process showed a bright and uniform surface in the darkfield optic image. Kamminga et al. [66] introduced a micromolding in capillaries (MIMIC) method similar to the imprinting method. Figure 8 show the differences between MIMIC and the imprinting lithography method (shown in Figure 8b) to fabricate micropatterns of perovskites. Instead of pressing the PDMS stamp for imprinting lithography, the MIMIC method utilizes capillary force to fill the perovskite precursor solution between the PDMS stamp and the substrate shown in Figure 8a. Compared with the film by spin-coating (shown in Figure 8c), the optical properties of the micropatterns by the imprinting lithography or MIMIC are stronger and enhanced [66].

### 5.2. Vapor Phase Deposition for Perovskite Crystals Arrays

**Figure 9:**
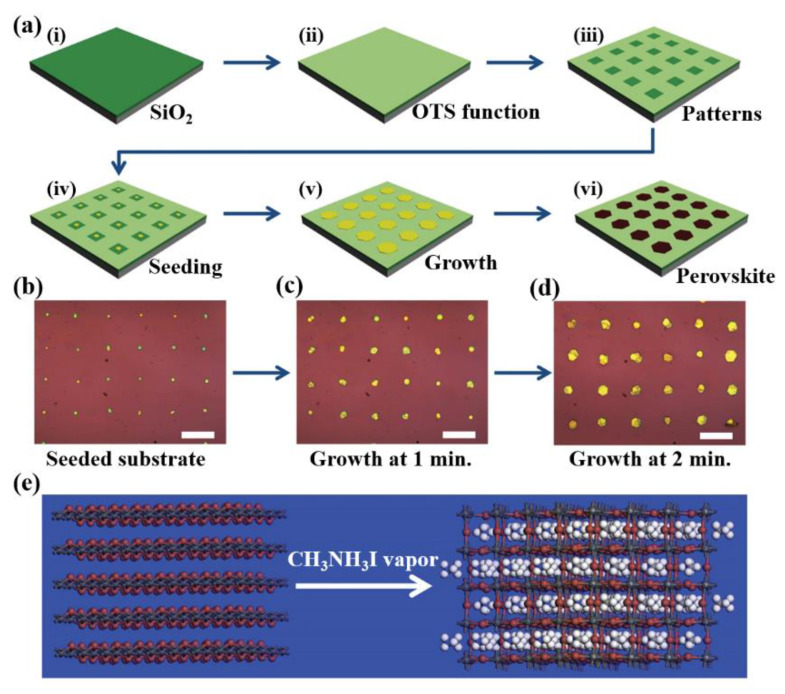
Schematic illustrations (**a**) the whole process of perovskite crystal arrays; (**a**_(**i**)_) cleaning a SiO_2_ wafer, (**a**_(**ii**)_) OTS deposition for hydrophobic surface formation by self-assembled method, (**a**_(**iii**)_) patterned, and peeled off the OTS for desired-patterned hydrophilic surface, (**a**_(**iv**)_) flowing an aqueous PbI_2_ solution to create PbI_2_ seeds only in hydrophilic areas, (**a**_(**v**)_) dip the surface with PbI_2_ seeds into saturated PbI_2_ solution for growth to a microplate, and (**a**_(**vi**)_) exposed the surface with PbI_2_ microplates into methylammonium iodide vapor to produce perovskite crystal arrays. (**b**) optical image of PbI_2_ seeds as prepared (**a**_(**iv**)_). (**c**,**d**) the optical images obtained after immersing the substrate with PbI_2_ seeds into saturated PbI_2_ solution for 1 min (**c**) and 2 min (**d**). Scale bars are 40 μm. (**e**) the transforming process from PbI_2_ layer to perovskite crystal by intercalating with CH_3_NH_3_I vapor. Reprinted with permission [67]. Copyright 2018, American Association for the Advancement of Science.

For perovskite crystal patterns fabrication, a directing method such as the blade coating, the inkjet, or several lithography methods is allowed precise control. On the other hand, the vapor phase deposition technique as an indirect method enables the formation of the perovskite patterns on a scalable substrate [67,68,69]. In 2015, Duan et al. [67] introduced a novel method for fabricating integrated device arrays with perovskite crystal growth, which is compatible with typical lithography. As shown in Figure 9a, the authors created a hydrophobic surface by depositing (octadecyl)trichlorosilane (OTS) on a silicon wafer in a self-assembled monolayer manner and then exfoliated the OTS surface in a regular arrangement using photolithography or electron beam lithography methods to revert the surface to hydrophilicity. By flowing the PI_2_ solution on the surface, PbI_2_ seeds were generated in the hydrophilic areas shown in Figure 9b, and no remains were left in the hydrophobic areas. The PbI_2_ seeds on the wafer were placed into a saturated PbI_2_ solution for growth (see Figure 9c,d). Then, the wafer with fully grown PbI_2_ seeds was placed in the tube furnace with methylammonium iodide vapor for the intercalation process to produce perovskite crystals (see Figure 9e). The PbI_2_ on the wafer were converted into CH_3_NH_3_PbI_3_ crystal arrays [67]. Later, other groups applied several other methods. For example, Dr. Yang’s group reported that cesium halide (CsX) arrays, as the first step of patterning on the wafer, converted perovskite arrays by flowing PbX_2_ vapor [68]. Shin et al. [69] employed poly(glycidyl methacrylate-r-2-((((2-nitrobenzyl)oxy)-carbonyl)amino)ethyl methacrylate) (poly(GMA-r-NBOCAEMA) as a photo cross-link polymer with preferred patterns on Si wafer and flow PbI_2_ vapor using chemical vapor deposition methods. The poly(GMA-r-NBOCAEMA) patterns were then converted into CH_3_NH_3_PbI_3_ crystal patterns.

## 6. Conclusions

Organic and inorganic halide perovskite crystals have received tremendous attention over time due to the high performance of their optoelectronic properties, which can open a new path to demonstrate state-of-the-art photovoltaic applications. Although major challenges to improve intrinsic and extrinsic instabilities of perovskites still remain, various studies for enhanced stability are reported, including encapsulating, doping, etc. [70,71]. In this review, various methods to fabricate micro and nanoscale patterns of halide perovskite crystals based on the coffee ring effects and Marangoni flow are described. The blade coating method (or called flow coating method) is a one-step way of forming a perovskite array film in one direction. As an advanced way or as a two-step way, the blade coating method with the perovskite precursor solution is applied to a substrate in which microchannels made by aqueous polymer nanoparticle suspension or the photolithography method are already placed. During solvent evaporation on the substrate, the perovskite precursor solutions are converted into perovskite crystal gratings with a homogeneous surface. Unlike the blade coating method, which is based on the coffee ring effect, the strategy of the inkjet printing method for perovskite crystal patterns with a uniform surface is the reduction of the coffee ring effect and the continuous convection current in a droplet using Marangoni flow. For this reason, researchers have studied methods such as adding polymers in the precursor solution to increase viscosity, controlling the temperature of the substrate to adjust evaporation rate, or using a confined area for the evaporation of droplets. In connection with the conjugation confined for the evaporation of the droplet solvent, the imprinting method was mentioned. Lastly, the authors have also discussed the vapor phase deposition method using surfaces with specific perovskite precursors capable of reacting with the vapors for the fabrication of perovskite crystals. Various patterns and arrays of halide perovskite crystals are available depending on the desired application devices in simple steps, at low cost, or over large areas with high throughput. 

## Data Availability

Not applicable.
